# Clinical rationale and safety of restaging transurethral resection in indication-stratified patients with high-risk non-muscle-invasive bladder cancer

**DOI:** 10.1186/s12957-018-1310-0

**Published:** 2018-01-15

**Authors:** Piotr Zapała, Bartosz Dybowski, Sławomir Poletajew, Łukasz Białek, Andrzej Niewczas, Piotr Radziszewski

**Affiliations:** Department of General, Oncological and Functional Urology, Medical University of Warsaw, Lindleya 4 Str, 02–005 Warsaw, Poland

**Keywords:** Bladder cancer, Cancer staging, Urologic surgical procedures

## Abstract

**Background:**

Indications for restaging transurethral resection of the bladder tumor (reTURBT) in patients with non-muscle-invasive bladder cancer (NMIBC) remain controversial. This study was aimed at evaluation of clinical value and safety of reTURBT in different clinical indications.

**Methods:**

This is a retrospective analysis of consecutive 141 patients who underwent TURBT followed by reTURBT in years 2011–2015 in a single department. Pathological results and surgical complications were analyzed in the whole study cohort and stratified by clinical stage (Ta, T1, Tx (no muscle in the specimen)) and grade (low-grade (LG), high-grade (HG)) of bladder cancer diagnosed at primary TURBT.

**Results:**

Full data was available for 132 patients. Residual disease was found in 53 patients (40.2%) with highest rate for Ta-HG cases (57.1%) followed by T1-HG (51.4%), Tx-HG (45.2%), T1-LG (32.1%), and Tx-LG (25.8%). In the multivariate analysis, high grade (*p* = 0.02) was the only independent predictor of residual disease. Upstaging to muscle-invasive bladder cancer was noticed in 9 patients (6.8%). The rate of grade ≥ 2 Clavien-Dindo complications (1.5 vs. 5.3%) did not differ significantly between TURBT and reTURBT cases.

**Conclusions:**

ReTURBT is a safe procedure that remains crucial for therapeutic and staging purposes in patients with T1, Tx, or high-grade bladder cancer found in the primary resection.

## Background

Approximately 70–80% of patients with bladder cancer initially present with a non-muscle-invasive disease (NMIBC). In these patients, transurethral resection (TURBT) with or without adjuvant intravesical chemo- or immunotherapy gives a chance for cure [1]. However, for stage T1 or high-grade (HG) tumors, there is a considerable risk of residual disease and/or understaging after initial TURBT. The risk of residual disease may be as high as 50–70%, while the understaging may affect even 20–30% of patients [2]. For this reason, many authors underline the need for restaging TURBT (reTURBT) in these specific clinical situations. The expert panel of the European Association of Urology (EAU) advises to perform reTURBT in all patients with T1 or HG tumors or when the detrusor muscle is lacking in the specimen (Tx). However, the need for reTURBT was recently questioned in patients with high-grade tumors and minimal or no invasion of the lamina propria, as well as in patients after TURBT performed by experienced urologists [3, 4].

The primary aim of this study was to assess the clinical value of reTURBT in patients stratified by clinical indications. The secondary aim of the study was to compare the safety of TURBT and reTURBT.

## Methods

### Patients

A group of consecutive patients with NMIBC who underwent reTURBT in years 2011–2015 in a single department were reviewed retrospectively. The indications for reTURBT were based on histopathological findings at the time of the initial TURBT. ReTURBT performed due to macroscopically incomplete TURBT was not defined as reTURBT, and patients who underwent such procedure were not included into the analysis. Patients with incomplete medical records, time interval between TURBT and reTURBT longer than 90 days, or suspected upper urinary tract malignancy were also excluded from the analysis.

Cancer-specific, demographic, pathological, and clinical data were investigated. Pathological data comprised of stage, grade, presence of concomitant *carcinoma* in situ (Cis), number of tumors, and presence of detrusor muscle in the specimen. Tumors were staged according to the 2009 TNM classification (stage Tx, Ta, and T1) and graded according to the 2004 WHO/ISUP classification (low-grade (LG) and high-grade (HG)). Clinical data included surgical and medical complications according to the modified Clavien-Dindo scoring system, length of catheterization, and length of hospital stay. All patients underwent upper urinary tract imaging at initial diagnosis (ultrasound or intravenous urography). The probability of recurrence and progression was calculated for each patient with the European Organization for Research and Treatment of Cancer (EORTC) calculator. Results of reTURBT were analyzed in the whole study group and after stratification by the following indications: Ta-HG, T1-LG, T1-HG, Tx-LG, and Tx-HG. Tx cases were subdivided into three groups: Tx(x), Tx(a), and Tx (1), where Tx(a) was defined as the absence of lamina propria invasion in a specimen lacking muscle, Tx (1) was defined as the presence of lamina propria invasion in a specimen without muscle, while specimens lacking both muscle and lamina propria were marked as Tx(x). Double indication for reTURBT was defined as Tx(a)-HG, T1-HG, or Tx(1)LG, and triple indication was defined as Tx(1)HG.

### Surgical technique

In the majority of cases, both TURBT and reTURBT were performed under spinal anesthesia with adequate premedication. General anesthesia was implemented only in individuals that did not accept or had contraindications for spinal block. The surgeries were carried out in a lithotomy position according to the protocol recommended by the EAU clinical guidelines. They consisted of bimanual palpation, urethral and bladder inspection, and resection of all visible tumors either in one piece for small papillary tumors (< 1 cm) or in fractions including the exophytic part and basal part and the edges of resected area for larger tumors. Specimens from different locations and fractions were sent to the pathologist in separate, labeled containers.

ReTURBT included deep resection of the scar and the edges of the initial resection site. Residual disease was defined as the presence of cancer in the reTURBT specimen. Random bladder or urethral biopsies were not routinely taken. Procedures were carried out by certified urologists or by supervised residents. In selected high-risk cases, narrow band imaging (NBI) was implemented at surgeon discretion. Photodynamic diagnosis (PDD) was not available.

### Statistical analysis

Continuous variables are presented as median or mean values accompanied by ranges or interquartile ranges (IQR). The differences between TURBT and reTURBT were evaluated with unpaired Student’s *t* test for continuous variables and with the chi-square test for categorical variables. For all statistical analyses, a two-sided *p* value < 0.05 was considered statistically significant. Statistical analyses were performed with the SAS System (version 9.4).

## Results

Among 141 patients who underwent reTURBT in 2011–2015, full pathological data was available for 132 patients (100 males, 32 females) aged 29 to 93 years with a median of 73 years (IQR = 16). Eighty-seven patients were diagnosed with the primary tumor, while 45 patients were treated due to bladder cancer recurrence. Indications for reTURBT were as follows: T1 disease in 63 patients, HG cancer in 73 patients, and stage Tx in 62 patients. Among the 62 patients with Tx, HG cancer was found in 31 patients (50%), invasion of lamina propria in 44 patients (71%), and LG cancer with no invasion into lamina propria in 8 patients (13%). Demographic and pathological data at the initial TURBT are summarized in Table 1.

**Table 1:** Demographic and pathological data (first resection)

Variable		*N* (%)	Mean (median)
Age			72 (73)
Sex	Female	32 (24%)	
	Male	100 (76%)	
Grade	LG	59 (45%)	
	HG	73 (55%)	
Stage	Ta	7 (5%)	
	Tx	62 (47%)	
	Tx(x)	6 (5%)	
	Tx(a)	12 (9%)	
	Tx(1)	44 (33%)	
	T1	63 (48%)	
Tumor size	< 30 mm	57 (55%)	
	≥ 30 mm	46 (45%)	
Number of tumors	Single	54 (47%)	
	2–7	44 (38%)	
	≥ 8	18 (16%)	
Disease history	Primary	80 (62%)	
	Recurrent	49 (38%)	
Probability of recurrence^a^	1 year		37% (38%)
	5 years		60% (62%)
Probability of progression^a^	1 year		7% (5%)
	5 years		20% (17%)

^a^Based on EORTC risk calculator for predicting recurrence and progression in patients with Ta and T1 bladder cancer

The median time interval between initial TURBT and reTURBT was 35 days (IQR = 19). In 33 patients (25%), the time interval was 6–12 weeks.

### Clinical value of reTURBT

Residual disease was found in 53 patients (40.2%). It was more likely in patients with HG than those with LG tumors (49.3 vs. 28.8%, *p* = 0.02). Tables 2 and 3 present detailed results of reTURBT stratified by indication.

**Table 2:** Results of reTURBT in all indications

		ReTURBT results			
ReTURBT indications	*n*	Any residual disease	Residual Ta or T1 disease	Muscle-invasive tumor	Cis
Ta-HG	7	4 (57%)	3 (43%)	0 (0%)	1 (14%)
T1-LG	28	9 (32%)	9 (32%)	0 (0%)	0 (0%)
Tx-LG	31	8 (26%)	3 (10%)	3 (10%)	2 (6%)
T1-HG	35	18 (51%)	12 (34%)	2 (6%)	4 (11%)
Tx-HG	31	14 (45%)	6 (19%)	4 (13%)	4 (13%)
TOTAL	132	53	33	9	11

*HG* high-grade, *T1* invasion of the lamina propria, *Tx* no muscle layer in the specimen, *LG* low-grade

**Table 3:** Results of reTURBT in various Tx indications

		ReTURBT result			
ReTURBT indication	*n*	Any residual disease	Residual Ta or T1 disease	Muscle-invasive tumor	Cis
Tx(x)–LG	3	0 (0%)	0 (0%)	0 (0%)	0 (0%)
Tx(x)–HG	3	1 (33%)	0 (0%)	0 (0%)	1 (33%)
Tx(a)–LG	8	2 (25%)	2 (25%)	0 (0%)	0 (0%)
Tx(a)–HG	4	1 (25%)	0 (0%)	1 (25%)	0 (0%)
Tx(1)–LG	20	6 (30%)	1 (5%)	3 (15%)	2 (10%)
Tx(1)–HG	24	12 (50%)	6 (25%)	3 (13%)	3 (13%)
Total	62	22	9	7	6

*Tx(x)* both detrusor muscle and subepithelial connective tissue lacking in the specimen; *Tx(a)* no intraepithelial invasion within available material, but detrusor muscle lacking in the specimen; *Tx(1)* intraepithelial invasion within available material and detrusor muscle lacking in the specimen; *HG* high-grade; *LG* low-grade

Table 4 presents the results of univariate analysis of risk factors of residual disease at reTURBT. In patients with single, double, and triple indications for reTURBT, residual disease was found in 34.9% (*n* = 15/43), 42.4% (*n* = 25/59), and 50% (*n* = 12/24) of cases, respectively.

**Table 4:** Univariate analysis of factors for residual disease at reTURBT

Variable		Residual disease (%)	OR (95% CI)	*p*
Age			1.0 (1.0–1.1)	NS
Grade	LG	29%	1	
	HG	49%	2.4 (1.2–5.0)	0.018
Stage	Ta	57%	1	
	Tx(x)	17%	0.2 (0.01–2.1)	NS
	Tx(a)	25%	0.3 (0.03–1.8)	NS
	Tx(1)	41%	0.5 (0.1–2.6)	NS
	T1	42%	0.6 (0.1–2.7)	NS
Tumor size	< 30 mm	33%	1	
	≥ 30 mm	43%	1.5 (0.7–3.4)	NS
Number of tumors	Single	39%	1	
	2–7	36%	0.9 (0.4–2.0)	NS
	≥ 8	44%	1.3 (0.4–4)	NS
Disease history	Primary	43%	1	
	Recurrent	37%	0.8 (0.4–1.6)	NS
Indications	Single*	35%	1	
	Double**	42%	1.3 (0.6–3.0)	NS
	Triple***	50%	1.9 (0.7–5.2)	NS

Six Tx(x) cases were considered as missing data in double/triple vs. single indication analysis

*reTURT* restaging transurethral resection of bladder tumor; *CI* confidence interval; *NS* not significant; *OR* odds ratio; *HG* high-grade; *LG* low-grade; *Tx(x)* both detrusor muscle and subepithelial connective tissue lacking in the specimen; *Tx(a)* no intraepithelial invasion within available material, but detrusor muscle lacking in the specimen; *Tx (1)* intraepithelial invasion within available material and detrusor muscle lacking in the specimen

*Single indication—Tx(a)LG, T1LG, TaHG

**Double indication—Tx(1)LG, Tx(a)HG, T1HG

***Triple indication—Tx(1)HG

Upstaging to muscle-invasive bladder cancer (MIBC) was diagnosed in 9 (6.8%) patients. Upstaging from Ta (Ta-HG) to T1 was not noticed.

### Safety of reTURBT

The overall postoperative complication rates were 1.5 and 5.3% for TURBT and reTURBT respectively. Grade 2 Clavien-Dindo complications were noticed in 1 and 3 patients after TURBT and reTURBT, respectively (0.8 vs. 2.3%, *p* > 0.05). Grade 3 or higher complications were noticed in 1 and 4 patients after TURBT and reTURBT, respectively (0.8 vs. 3.0%, *p* > 0.05). After TURBT, hematuria requiring another transurethral intervention occurred in one case and urine retention also in one case, while after reTURBT, hematuria requiring transurethral intervention occurred in two cases, bladder perforation requiring cystorrhaphy in two cases, atrial fibrillation requiring pharmacological cardioversion in one case, and urine retention in two cases. Mean length of a postoperative hospital stay was longer for TURBT than for reTURBT (2.9 vs. 2.4 days, *p* = 0.02). A trend for a longer catheterization time after TURBT than reTURBT was observed (1.8 vs. 1.5 days, *p* = 0.07).

## Discussion

As there are controversies regarding indications for reTURBT, we intended to compare significance of particular indications and evaluate them as the predictors of residual disease. Moreover, little is known about the safety of reTURBT, which we intended to analyze as a secondary study aim. Considerably high rate (40.2%) of residual tumor was found in reTURBT, which is consistent with the findings from some previously published studies [2, 5, 6]. Our report is another argument for a routine reTURBT in T1 tumors, high-grade cancer, and when a detrusor muscle is lacking in an initial specimen.

The strategy of reTURBT remains under the debate. Kamat directly calls it a failure of urological technique, which by definition assumes the risk of incompleteness [7]. Also, the indications for reTURBT are not clear and vary between institutions and authorities. Expert panel of the European Association of Urology recommends reTURBT in patients with T1 tumors, high-grade cancer, if there is no muscle layer in the specimen and after incomplete initial TURBT [1]. However, the evidence behind this recommendation is not consistent. In the recent update of EAU guidelines, lack of muscle in specimen without evidence of T1 or HG has been questioned as indication to restaging resection in recurrent tumors. Since this retrospective study was based on patients treated before this update, we decided not to remove pTx(a)LG patients from analysis. Noteworthy, although no restaging to MIBC was recorded in this group, residual disease was found in 2 patients. These findings emphasize that even in low-risk tumors, surveillance on resection completeness is of primary importance.

For patients with T1 tumors, it was shown that reTURBT improves staging accuracy [8]. Kulkarni et al. outlined that reTURBT was aimed not only at proper staging but also at clearance of residual disease and prognostication [9]. Improved response to BCG therapy is an advantage value of reTURBT [10]. Finally, Divrik et al. in a prospective randomized trial clearly showed that reTURBT increases the recurrence- and progression-free survival in patients with T1 tumors [11]. Conversely, Sfakianos et al. in a retrospective analysis found that no reTURBT performed is a stronger predictor of recurrence than the stage or grade of cancer [12]. However, the evidence concerning the value of reTURBT in patients with Ta HG tumors is lacking. Thus, the recommendation of reTURBT in this group of patients is not universal and seems to extrapolate findings from T1 HG patients. A consensus document endorsed by the Canadian Urological Association states that reTURBT in patients with Ta HG tumors is not routinely recommended [3]. In turn, expert panel of the American Urological Association suggests to consider reTURBT in this group of patients, however, as a grade C recommendation [13].

Value of reTURBT in T1 HG patients has recently been questioned as well, especially when the muscle is present or when a procedure is both complete and performed by an experienced urologist. In a large retrospective observational study, Gontero et al. found that reTURBT was not beneficial in patients with T1 HG tumors and muscle sampled at TURBT [4]. For this reason, a consensus document endorsed by the Canadian Urological Association advises to perform reTURBT in T1 tumors when the muscle was not sampled, while in the cases of small T1 lesions, it can be omitted if the experience of the surgeon is adequate [3]. The issue of surgical experience and its measurement is both controversial and debatable. Zurkirchen et al. found that surgical experience has no effect on the risk of residual tumor at reTURBT and concludes that reTURBT is also a must for experienced urologists [14]. In our group, we could not draw any conclusions on the effect of experience as the retrospective design would cause insufficient numbers of patients in each of the subgroups. However, it is our practice that resections of extensive tumors and most of reTURBTs are performed by experienced surgeons.

In our material, relatively low portion of patients were upstaged to MIBC after reTURBT, while literature data present the rate of 5–40% [2, 6, 15, 16]. Selection of patients for reTURBT and significant number of pTxLG, as well as the quality of initial resection, might be possible reasons for differences between results in published studies. We believe that proper technique of the primary resection and instant MIBC diagnosis are the main explanation for low upstaging rate in our material. Since some of bladder tumors show tentacular growth pattern, providing deep and wide resection is crucial in improving the quality of initial TURBT. As illustrated by Herr and Donat [17], accurate assessment of pathological extent can be often achieved only after extending resection margin even by an additional of 2–3 cm. Deep resection out to 4 cm of surrounding radius provides removal of invisible lesions like CIS and finger-like submucosal invasion foci. In terms of depth and extension, precise sampling might also be of importance. Particular sampling methods can be used to ensure complete tumor resection and provide histopathological “self-control” [18].

Low rate of upstaging prevented us from analyzing risk factors and interpretation of its occurrence in subgroups. Although eight out of nine MIBC cases were detected after finding subepithelial invasion in TURBT, special attention should be paid to a single case of Tx(a)-HG. This example shows that even if no evident invasion behind lamina propria is found in initial resection, muscle-invasive disease can be missed if the material is diagnostically insufficient in HG tumors.

Noticeably, high rate of residual disease in the reTURBT specimens, especially in T1 tumors and also in prognostically favorable Ta, raises the question about possible technical improvements. Similar residual rates were observed by Richterstetter et al. [19]. Authors supported application of differentiated TURBT technique and use of fluorescence-guided resection using photodynamic diagnosis.

Although the experience of a surgeon has unclear influence on a risk of residual disease [14], less experienced urologists are more likely to perform TURBT without providing the presence of detrusor muscle in specimen which can affect long-term prognosis [20, 21]. Improvement of TURBT technique and learning facilitation can be however efficiently achieved by institutional standardization of a resection protocol. In a recent prospective study, an Italian group proved that implementation of institutional quality control program based on direct pathologist-urologist cooperation decreases the rate of stage Tx diagnosis [22].

Risk factors for residual tumor in reTURBT may include presence of Cis, multiple tumors, stage and grade of tumor, size of tumor, interval between two resections, recurrence history, surgeon experience, and others [5, 6, 14, 23, 24]. Their predictive values vary noticeably between studies. In our study, the only significant predictor of residual disease in reTURBT was HG tumor. We were not able to confirm other previously proposed predictors, but considering variability observed in literature, it can only indicate complex nature of predicting residual disease at reTURBT. We suspect that the reason for observed phenomenon might be a low rate of upstaging reported in our series, as the majority of described predictive factors correlates with occurrence of muscle-invasive cancer.

In our material, we observed high incidence of Cis in patients with HG tumors. In the contemporary series of 2415 patients with T1G3 tumors, Gontero et al. found concomitant foci of Cis in as many as 24% of cases, which increases to 34% in a subgroup of patients with recurrence [25]. Moreover, Hara et al. noticed concomitant Cis in 59% of high-risk NMIBC patients, in the vast majority predicted by positive urine cytology [26]. Also, the risk of Cis in the prostatic urethra is considerably high, as reported by Palou et al. [27]. While high-grade cancer is considered a risk factor for the presence of Cis, question about random biopsy application in these cases can be raised. Implementation of PDD/fluorescence cystoscopy [19, 28], narrow band imaging cystoscopy [29], or extended reTURBT approach [19] might also be justified in this group of patients.

Our study shows that the safety of TURBT and reTURBT is comparable. As deep resection remains crucial in reTURBT, the trend to more frequent complications after reTURBT, especially bladder perforation, is not surprising. On the other hand, length of hospital stay and catheterization were significantly longer after TURBT. This could be caused by many technical differences between TURBT and reTURBT and/or anesthesia time, affecting the time to return of voiding function. In recent meta-analysis, only two randomized controlled trials reporting significant complications were found [30]. Similarly, to our records, they included bleeding requiring coagulation (3.7 and 3.8% patients respectively), transient urinary retention (1.2 and 1.9% patients respectively), and epididymitis (1.2 and 1.9%, respectively).

Small size of the study population and local character of the analysis are the main limitations of our retrospective study. They may result in some institution-specific observations. Low prevalence of upstaging and presence of Cis in initial resection observed in our department indicate the risk of selection bias. Despite these limitations, our study is the first analysis focused on comparing clinical importance of reTURBT indications. In opposite to the previous reports, the only significant risk factors of residual disease in reTURBT are high-grade tumor at initial resection and the presence of two indications simultaneously. Most importantly, we present another report with a high prevalence of residual disease in reTURBT performed in high-risk NMIBC patients, which strongly supports its routine implementation. The presence of HG tumor in TURBT or multiple indications for reTURBT (T1-HG, Tx-HG) should always be managed with awareness of exceptional risk of residual tumor. Moreover, in patients with HG tumors, the risk of concomitant Cis should be taken into consideration. In these individuals, extensive resection should be performed with random multisite cold cup biopsy of the normally looking mucosa. Establishing which patients are at the highest risk of muscle-invasive cancer or invisible residual disease could identify potential candidates who benefit from modern cystoscopy tools or radical cystectomy.

## Conclusions

ReTURBT is a safe procedure that improves the staging of bladder cancer and increases the chances for complete cancer ablation. It should be considered in all patients with stages T1, Tx, or high-grade bladder cancer.

## Abbreviations

EAU: The European Association of Urology
HG: High-grade
LG: Low-grade
MIBC: Muscle-invasive bladder cancer
NMIBC: Non-muscle-invasive bladder cancer
reTURBT: Restaging transurethral resection of the bladder tumor
TURBT: Transurethral resection of the bladder tumor
